# Slow and steady wins the race: an examination of bacterial persistence

**DOI:** 10.3934/microbiol.2017.2.171

**Published:** 2017-03-27

**Authors:** Tara L. Renbarger, Jennifer M. Baker, W. Matthew Sattley

**Affiliations:** Division of Natural Sciences, Indiana Wesleyan University, Marion, Indiana 46953, USA

**Keywords:** bacterial persistence, persister cell, toxin-antitoxin module, antibiotic resistance, biofilm

## Abstract

Bacterial persistence is a state of metabolic dormancy among a small fraction (<1%) of a genetically identical population of cells that, as a result, becomes transiently resistant to environmental stressors. Such cells, called persisters, are able to survive indeterminate periods of exposure to challenging and even hostile environmental conditions, including nutrient deprivation, oxidative stress, or the presence of an antibiotic to which the bacterium would normally be susceptible. Subpopulations of cells having the persister phenotype is also a common feature of biofilms, in which limited space, hypoxia, and nutrient deficiencies all contribute to the onset of persistence. Microbiologists have been aware of bacterial persistence since the early days of antibiotic development. However, in recent years the significance of this phenomenon has been brought into new focus, as persistent bacterial infections that require multiple rounds of antibiotic treatment are becoming a more widespread clinical challenge. Here, we provide an overview of the major features of bacterial persistence, including the various conditions that precipitate persister formation and a discussion of several of the better-characterized molecular mechanisms that trigger this distinctive mode of bacterial dormancy.

## 1. The Discovery of Bacterial Persistence

Since their discovery in 1928, antibiotics have been used to treat numerous bacterial infections and have saved millions of lives [1],[2]. However, bacteria have engaged in their own “arms race” by rapidly developing ways to combat antibiotic efficacy and prevent being killed by these chemotherapeutic agents. In 1940, Abraham and Chain provided the first description of an antibiotic-resistance mechanism [3]. Since then, antibiotic resistance and bacterial persistence have been major drivers of a growing problem in clinical and veterinary medicine, as many pathogenic bacteria are resistant to one or more antibiotics, and “pan-resistant” bacteria that cause untreatable infections are becoming more common. Resistant pathogenic strains of bacteria cause more than 23,000 deaths each year in the United States alone [2].

**Figure 1.**
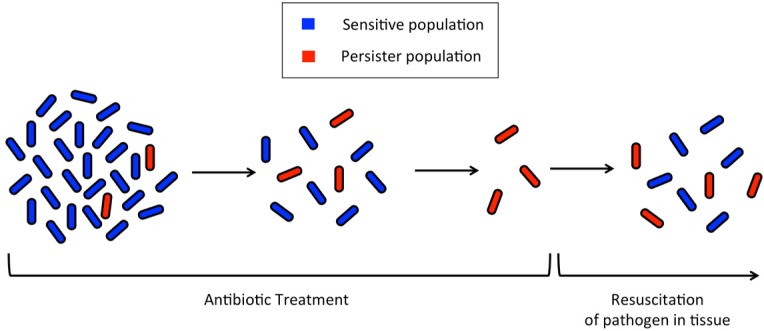
Progression of persister cell formation during antibiotic treatment. At the onset of antibiotic exposure, the total cell population contains a small subset of persister cells that maintain a dormant but viable state. At the completion of the antibiotic regimen, susceptible cells are destroyed, but persister cells remain and are able to resuscitate, propagate a new population of cells, and cause recurrent disease.

The concept of bacterial persistence and antibiotic resistance dates back to over 75 years ago when Joseph Bigger treated a staphylococcal culture with penicillin. Bigger observed that not all of the bacteria in the culture were killed by the treatment, but rather, some of the cells persisted in a viable state (Figure 1) [4]. Later studies by Moyed and Broderick demonstrated that the persistence of some cells is a phenotype, and this phenotype is not permanent; bacterial cultures started from persister cells were sensitive to the same antibiotics to which the persister parents were resistant [5]. The exact mechanism by which persister cells resist the antibacterial effects of antibiotics has not yet been fully elucidated. However, recent studies have unveiled several cellular pathways, discussed below, by which these persister populations arise.

## 2. Distinguishing Bacterial Persistence from Resistance

Because their modes of action are generally dependent upon interfering with various enzyme-mediated, biosynthetic mechanisms in their target cells, antibiotics typically require actively growing cells to achieve their antibacterial effect. Compared to normally dividing, vegetative cells, persister cells are metabolically dormant and are, therefore, resistant to being killed by antibiotics, rendering antibacterial drugs ineffective on these cell types. An interesting facet of persister cells is that they are genetically identical to non-persister cells, whereas other types of antibiotic-resistant bacterial cells are the result of an altered genotype due to the acquisition of new antibiotic resistance genes. Bacteria can acquire these antibiotic resistance genes via either large genetic exchanges between cells, such as by the horizontal transfer of plasmids or other mobile genetic elements containing resistance factors (e.g., the *mcr-1* gene, which can cause bacteria to become resistant to the last-resort drug colistin [6]) or through the sequential accumulation of smaller genetic changes (e.g., the 35 point mutations that caused a wild-type strain of *Staphylococcus aureus* to become vancomycin-resistant [7]). An antibiotic resistance gene database estimates that 23,137 genes account for 380 types of antibiotic resistance to 249 different antibiotics [8].

## 3. Differentiation into a Persister Cell and Quantifying Persisters

Within a microbial population, a small subpopulation will spontaneously exhibit the persistence phenotype. This is a reversible change that allows bacteria to switch to and from the persister state to the normal growth state [9]. A 2005 study by Kussell et al., suggested that some large populations of bacteria employ persister cells as an “insurance policy” against antibiotic exposure [9]. These persister cells raise the overall fitness of the population by developing phenotypic differences within an isogenic population; the slow growth and division rates of persister cells allow them to be more resilient to antibiotics and other detrimental environmental changes than their normally growing counterparts (Figure 1) [9],[10]. The persistence phenotype exhibited by the small subpopulation allows for the majority of cells in the population to grow at the fastest rate possible under the prevailing conditions. At the same time, the persister cells maintain a minimal metabolism, essentially becoming dormant but remaining viable, so as to provide the greatest chance for survival of the population if environmental conditions become inhospitable.

The subpopulation of persister cells within the larger bacterial population is typically very small, usually about 0.01% or less of the total viable population, but this percentage can vary widely depending on the species and the prevailing environmental conditions. Most of the studies that have analyzed a particular gene's role in persistence have been performed in *Escherichia coli*, in which the number of persister cells within a wild-type exponential population is typically in the range of 10^−7^–10^−5^ [5],[11],[12]. Under similar growth and sampling conditions, “high persistence” *E. coli* mutants, such as the *hipA7* mutant, can generate 10^−3^–10^−2^ persisters (up to 1% of total cells) [12]. A study of persistence in the difficult-to-control pathogen *Pseudomonas aeruginosa* found that the percentage of persister cells in the population varied considerably depending on whether the cells were planktonic, in which case persisters consisted of 0.001% of the total population, or immobilized in a biofilm, where persisters comprised as much as 0.1% of the population [13],[14]. This observation may be linked to a propensity for stationary-phase, biofilm-associated cells to more readily assume the metabolically dormant phenotype characteristic of persistence [14].

The conventional method for measuring persistence is to grow the bacterial cultures to the desired stage (e.g. stationary phase) at which persistence will be evaluated [15],[16]. At that time, samples of cells are removed to determine the original number of bacteria present in the pre-treated culture, and then the bacteria are incubated with a lethal dose of an antimicrobial agent (e.g. ampicillin), which should kill active bacteria but leave the persister cells unaffected. Next, culture samples are washed and serially diluted before being plated. After an overnight to two-day incubation at 37 °C, colonies are counted and calculations are made to determine the number of persister cells compared to the total number of cells in the original culture. Several factors in these methods influence the frequency of persisters. The length of time of antimicrobial exposure, dosage of the antimicrobial, and even the recovery medium used (e.g., recovering persisters on broth versus agar media) influence the percentage of persisters within the sample [16],[17].

Traditionally, the success of persisters against antibiotic treatment has been attributed to persister dormancy prior to antibiotic exposure. However, Orman and Brynildsen used fluorescence-activated cell sorting (FACS) to show that persister cells can develop from rapidly growing cells in the absence of antibiotic exposure [18]. Although dormancy is a prerequisite for persistence, it is important to recognize that a bacterial cell found in a dormant state does not necessarily make it a persister or guarantee that it will survive antibiotic exposure [18]. Indeed, out of the dormant subpopulations sampled in the study, less than 1% of cells were persisters [18]. Collectively, these evidences suggest that persistence is not merely a result of dormancy, but that a more complex, and as yet unknown, underlying mechanism exists.

In addition to stochastic mechanisms causing normal cells to become persister cells, responsive mechanisms also play a role in the formation of the persister phenotype. Persister cells can form in response to environmental stressors, such as conditions of extreme pH, high temperature, and the presence of oxidative stress [19]. To survive transient hostile conditions, some of the bacteria in the population enter into a dormant phase with low metabolic activity. After the stressful environmental conditions subside, the persister cells can revert back to normal growth and division. We explore the key molecular signaling mechanisms that control this phenotypic exchange in later sections.

## 4. Factors Affecting Persister Formation

### 4.1. Growth phase

**Figure 2.**
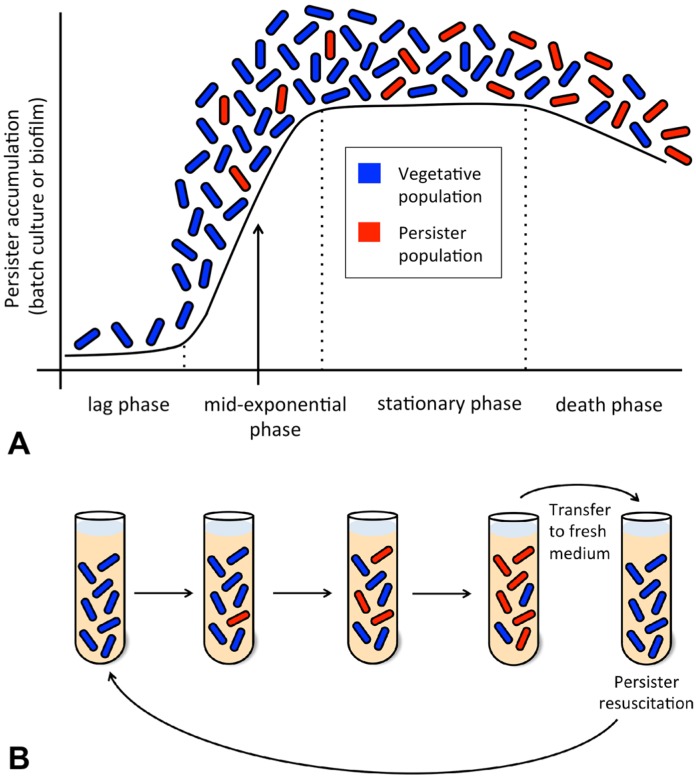
Progression of the persister phenotype in batch cultures and biofilms. (A) A typical growth curve as might be observed in a batch culture or biofilm is shown. Note that persistence first develops in a small subpopulation of cells during the mid-exponential phase of growth and is well established by the stationary phase. (B) The progression of a batch culture is shown, with tubes corresponding to the growth phases in part A. Upon transfer to fresh medium (or upon dispersal away from a biofilm), persisters encounter more favorable environmental conditions and can resuscitate to propagate a new population, analogous to the scenario in Figure 1.

The number of persisters in a growing population varies depending on the phase of growth, with the highest percentage of persisters found at stationary phase [14]. Persisters are typically absent in the early exponential phase of growth, but by mid-exponential phase, persisters begin to appear in the population, and a maximum of approximately 1% is reached during stationary phase (Figure 2A) [13],[14],[20]. The percentage of persister cells present in each growth phase varies among different species of bacteria. Some bacteria produce a relatively low quantity of persisters, whereas stationary phase cultures of, for example, *P. aeruginosa* have high numbers of persisters (10^−2^ to 10^−3^) and have similar antibiotic tolerance levels compared to that of *P. aeruginosa* found in biofilms [13],[14],[21],[22].

In addition to species variations, the percent composition of persisters in a batch culture is dependent upon the age of the inoculum, as well as the composition of the medium in which the bacterium has been grown [23]. When stationary phase bacteria are diluted into fresh medium, a subset of the population will begin to grow immediately whereas other cells grow later, or not at all (Figure 2B) [24],[25],[26]. Some believe that the varying rates in growth resumption are part of a strategy that bacteria use to increase fitness; bacteria that are rapidly recovering are more vulnerable to being killed by antibiotics or sudden environmental stresses compared to the dormant cells [12],[13],[27]. Studies by Luidalepp et al. demonstrated that a younger stationary-phase inoculum results in lower frequency of persister cells than that of an older stationary-phase inoculum [23]. Thus, the bacteria taken from an older stationary-phase culture were more resistant to various antibiotics than their younger counterparts [23].

Hosts can provide sufficient nutrients for bacterial populations to continue to grow, whereas nutrient depletion occurs during in vitro studies, resulting in limitations on studying persister development [28]. Therefore, in vitro studies using nutrient-starved stationary phase bacteria would not be the optimum way to mirror in vivo persisters where nutrients can be abundant. A recent model was developed to study persistence in a nutrient-rich environment. This model employs nutrient shifts, a form of metabolic stress, and by affecting the carbon metabolic flux was shown to produce a large number of persisters [29],[30]. Studies by Radzikowski et al. examined both nutrient-shift generated persisters and starvation-generated persisters to determine phenotypic and proteomic similarities [31]. They concluded that RpoS (σ^S^), a sigma factor active during starvation and stress responses, drove the stress response and the proteomic profile of both nutrient-rich and nutrient-starved persister types. In addition, increased levels of (p)ppGpp (guanosine tetraphosphate and guanosine pentaphosphate) were observed in both types of persisters [31]. Upregulated σ^S^ levels can be caused by increased (p)ppGpp levels [32].

### 4.2. Biofilms

Biofilms constitute a physical barrier against host defenses, including antibodies and immune cells, and antibiotic efficacy. Biofilms are a primary culprit responsible for recalcitrant infections (Figure 1), and most microbial pathogens are capable of forming biofilms [14],[22],[33],[34]. Biofilms are notoriously difficult to treat with antibiotics, a phenomenon traditionally believed to be due to the physical structure of the microbial population; the dense, surface-associated cellular composition and thick extracellular matrix impede penetration of the drugs. However, some antibiotics do penetrate biofilms, and recent studies have revealed that an accumulation of persisters also contributes significantly to the antibiotic tolerance of biofilms [13],[35]. Spoering and Lewis found that biofilms have 100–1,000 fold more persisters than planktonic cultures [13]. These persisters have the potential to resuscitate, resume active growth, and disperse away from the biofilm given the proper conditions, which will vary among different species.

## 5. Cellular Signaling Mechanisms Contributing to Persister Formation

### 5.1. Secondary messenger (p)ppGpp

The major chemical signal for the onset of persistence is the production and accumulation of the alarmone (p)ppGpp, a regulatory nucleotide through which cells alter their metabolism in response to stress. The stringent response and other environmental stressors trigger the production of (p)ppGpp by the activities of RelA and/or SpoT. RelA associates with the large subunit of the ribosome and produces (p)ppGpp by catalyzing the transfer of two phosphate groups from ATP to either GTP or GDP. The docking of an uncharged tRNA molecule to the aminoacyl (A) site of the ribosome, as may occur during nutrient-starved conditions, triggers this reaction. SpoT, which helps suppress the stringent response by degrading (p)ppGpp when environmental conditions are favorable, reverses its activity and synthesizes (p)ppGpp in poor growth conditions, especially during periods of nutrient deprivation.

Through the accumulation of (p)ppGpp, the cell is able to downregulate DNA replication and protein synthesis, as well as modify transcription levels and halt cell division mechanisms. Transcriptional modification via (p)ppGpp occurs through its direct interactions with RNA polymerase and by activation of RpoS (σ^S^) and RpoE (σ^E^). RpoS and RpoE are stress response sigma factors that are activated, respectively, during stationary phase and in response to extracytoplasmic stress that leads to misfolding of periplasmic proteins [36]. Whereas persisters typically form from cells in stationary phase when (p)ppGpp production is upregulated, rare cells can also activate (p)ppGpp during exponential growth [37]. In addition, the development of biofilms stimulates the production of (p)ppGpp, primarily through nutrient deprivation, including hypoxic conditions.

### 5.2. The HipBA toxin-antitoxin module

**Figure 3.**
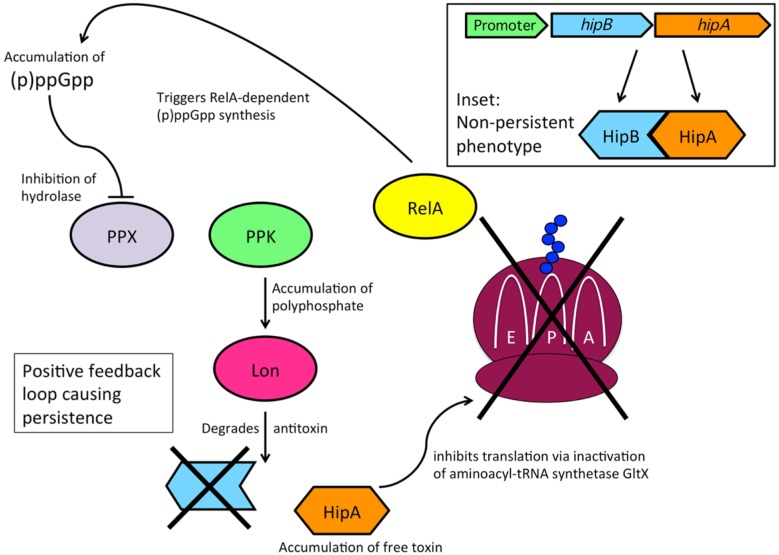
Mechanism of the HipBA toxin-antitoxin module. The *hip* operon encodes the Glu-tRNA synthetase (GltX) toxin HipA, as well as the antitoxin HipB. In cells that are growing and dividing, HipB-bound HipA is inactive, and translation occurs normally. In persister cells, elevated levels of (p)ppGpp inhibit polyphosphate hydrolase (PPX), leading to higher levels of polyphosphate in the cell due to the activity of polyphosphate kinase (PPK). Polyphosphate activation of Lon protease causes HipB degradation and liberation of HipA, which leads to the binding of uncharged tRNAs at the aminoacyl (A) site, ribosome stalling, and increased production of (p)ppGpp by activated RelA.

In 1983, Moyed and Bertrand isolated high-persistence (*hip*) *E. coli* mutants, which showed that persistence could be attributed to genetic factors [11]. Studies since then have revealed that toxin-antitoxin (TA) pairs, or modules, are largely responsible for the genetic basis of persister formation due to their induction of a dormant state, which allows cells to tolerate exposure to antibiotics and other harsh conditions [20],[38],[39]. TA modules consist of a pair of genes—one gene encoding a toxin, and the other gene encoding its complementary antitoxin—within an operon. The genes *hipB* and *hipA* encode the HipBA TA module; HipA is a stable toxin, whereas HipB is an unstable antitoxin (Figure 3) [12]. The *hip* operon contains a regulatory region having −35 and −10 promoter elements, as well as inverted repeat sequences and an integration host factor consensus-binding site; downstream of this region is the short open reading frame (ORF) *hipB* followed by the longer ORF *hipA* [40].

Multiple studies have investigated the roles of *hipA* and *hipB* within the context of bacterial persistence and found that the *hip* operon is autorepressed by the antitoxin HipB, which functions as a DNA-binding protein and is also able to neutralize the HipA toxin by forming a tight complex with free HipA during normal growth conditions [41],[42]. During periods of cellular stress, HipB is degraded by the protease Lon, which liberates HipA toxin from its antitoxin (Figure 3) [43]. In this unbound state, the HipA toxin functions as a kinase that was originally thought to phosphorylate EF-Tu (elongation factor thermo unstable) but has now been shown to target the active site of Glu-tRNA synthetase (GltX) [42],[44]. GltX inhibition by HipA results in an increased concentration of uncharged glutamyl tRNAs (tRNA^Glu^). Whereas RelA is bound to the ribosome in an inactive state under normal conditions, the accumulation of uncharged tRNA^Glu^ disrupts ribosomal function, causing the release and thus activation of RelA, which triggers RelA-dependent synthesis of (p)ppGpp (Figure 3) [44],[45] . Elevated levels of (p)ppGpp inhibit polyphosphate hydrolase (PPX), causing an accumulation of polyphosphate by the activity of polyphosphate kinase (PPK) (Figure 3); this activates the previously mentioned Lon protease and perpetuates the dormant state.

Deletion of the *hipBA* operon causes a sharp decrease in the frequency of persister formation during stationary phase (Table 1) [46]. By contrast, certain mutations introduced into *hipA* have been shown to reduce the affinity of the toxin to the antitoxin, which increases activity of the toxin and favors persister formation (Table 1) [46]. For example, a mutant strain of *E. coli* containing *hipA7* (a modified *hipA* gene having two point mutations that presumably cause HipA to interact poorly with the antitoxin) had a 10–10,000 fold higher frequency of persister cell formation compared to wild type [12],[14],[23],[42]. Increased persister frequency can also result from overexpression of *relA*, which leads to increased levels of (p)ppGpp [12]. In contrast, deletion of *relA* in the *hipA7* mutant caused reduced persistence, and the deletion of both *relA* and *spoT* eliminated the persistence phenotype altogether [12]. In other studies, overexpression of *hipA* in *E. coli* boosted persister development 10–1000 fold over wild type [14],[48], and experiments using *hipB*-disrupted or *hipB*-deficient strains of *E. coli* showed an increase in persister frequency from 10^−5^ to 10^−3^ [40]. Table 1 provides a summary of the influence of expression levels of key genes on development of the persister phenotype.

### 5.3. Messenger RNA (mRNA) endonuclease TA modules

RelE and DinJ are TA module elements linked to dormancy and were identified in an expression profile of persisters [49],[50]. RelE toxin is an mRNA endonuclease that inhibits translation, and therefore it has been linked to increased tolerance to antibiotics that affect ribosomal functioning, such as macrolides and tetracyclines [47],[49],[51],[52]. When RelE is overproduced in *E. coli*, there is a drastic increase in persister formation [47]. However, when *relE* is deleted, there is no observed difference in persister formation compared to wild type [50]. The toxin-encoding gene *yoeB*, which is expressed in wild-type persister cells of *E. coli*, is a homolog to *relE*, and as observed with the *relE* deletion, deletion of *yoeB* had no effect on persister formation (Table 1) [53]. This suggests that multiple mechanisms for inducing persister formation exist in *E. coli*, and this may also be true for other species of clinical significance, such as *Mycobacterium tuberculosis* and *Salmonella enterica* [54].

YafQ and DinJ also comprise a TA system present in *E. coli*. The YafQ toxin alters metabolism through its mRNA endonuclease activity that specifically targets in-frame 5′-AAA-G/A-3′ sites in TnaA transcripts [55]. The *tnaA* gene encodes tryptophanase, which converts tryptophan into indole (plus pyruvate and NH_3_). Through an unknown mechanism, the presence of indole, a common bacterial signaling molecule [56], suppresses persistence, and it has been shown that the addition of indole to cultures of *E. coli* decreases persister frequency [55]. By cleaving TnaA mRNA transcripts, YafQ is able to increase persistence by reducing levels of tryptophanase, and therefore also reducing levels of indole [55].

MazF, like RelE and YafQ, is an mRNA endonuclease toxin that, in coordination with MazE, comprises another TA module identified in *E. coli*. MazF toxicity is dependent upon the production of (p)ppGpp [57]. Similar to *relE*, overexpression of *mazF* increases persister formation, whereas the knockout of *mazF* causes no phenotypic change (Table 1) [53].

**Table 1.** Influence of gene products on persister phenotype.

Effect on Persistence	Gene	Basis	Reference
*Increased persistence*	*hipA*	overexpression	47
	*hipA7*	2 point mutation	12
	*hipB*	no expression	40
	*relE*	overexpression	47
	*mazF*	overexpression	53
	*relA*	overexpression	12
	*glpD*	overexpression	12,50
	*dnaJ*	overexpression	65
	*pmrC*	overexpression	65
*No effect on persistence*	*relE/yoeB*	no expression	53
	*mazF*	no expression	53
	*ygiU*	no expression	53
*Decreased persistence*	*hipBA*	no expression	46
	*relA*	no expression	12
	*relA* + *spoT*	no expression	12
	*phoU*	no expression	62
	*ubiF*	non-functional mutant	63
	*sucB*	no expression	23
	*ygfA*	no expression	23
	*glpD*	no expression	50
	*plsB*	attenuated mutant	50
	*dnaJ*	no expression	23
	*glpC*	no expression	23
	*surA*	no expression	23

### 5.4. Other TA modules and persistence elements

A comparison of the gene profile of persisters and nonpersisters in biofilms revealed high levels of expression of *ygiU* (*mqsR*), which was predicted to be a cyanide hydratase and is induced upon biofilm formation [58],[59]. The toxin-encoding *ygiU* is part of a two-gene operon that also includes *ygiT* (*mqsA*), which encodes the antitoxin and is a transcriptional repressor [60]. Gonzalez Barrios et al. described *ygiU* as a global regulator controlling biofilm formation [60]. Interestingly, *ygiU*-deleted strains showed no phenotypic effect on the frequency of persister formation; however, in some strains a decrease of biofilm formation and abrogated motility was observed (Table 1) [53],[61].

The gene *phoU*, which controls the phosphate uptake system *pstSCAB*, was reported to function in persistence by responding to environmental changes, especially nutrient availability [62]. Li and Zhang proposed that PhoU is a global negative regulator that contributes to repression of cellular metabolism by downregulating genes and proteins necessary for energy production, membrane transport, and motility [62]. Strains of *E. coli* with deleterious *phoU* mutations maintained a hyperactive state, as evidenced by higher expression of energy metabolism genes, membrane transporters, and motility genes. Hyperactive *phoU* mutants were more susceptible than the parental strain to multiple antibiotics and environmental stresses. In addition, the *phoU* mutants formed fewer persisters than the parental strain [62]. However, Luidalepp et al. reported that this phenotype was observed only when the initial inocula were obtained from older (late stationary-phase) cultures [23].

*E. coli* strains having mutated *ubiF* (a gene encoding an enzyme involved in ubiquinone biosynthesis) and *sucB* (a gene encoding a key enzyme involved in the tricarboxylic acid cycle) genes have been shown to be deficient in energy production [63],[64]. These mutants were found to negatively affect persister survival by demonstrating higher susceptibility to antibiotics, oxidative stress, and decreased pH [63]. Ma et al. speculate on the energy demand for persister bacteria to survive stressful conditions, and the potential inability of *ubiF* and *sucB* mutants to meet minimal energy needs would result in persister bacteria being more susceptible to stresses [63]. In addition, *sucB* and *ygfA* (5-formyltetrahydrofolate cyclo-ligase) deletions in *E. coli* showed decreased persister levels regardless of whether the inocula were obtained from fresh or aged cultures [23]. Furthermore, an attenuated mutant of *plsB* (*sn*-glycerol-3-phosphate acyltransferase), a gene that is also associated with energy metabolism, showed a decreased level of persister formation [50].

*glpD* (*sn*-glycerol-3-phosphate dehydrogenase) is part of the *glp* regulon in *E. coli*, which consists of five operons associated with glycerol degradation and energy metabolism. The upregulation of *glpD* has been observed in a transcriptional profile of isolated persister cells [53]. The overexpression of *glpD* in *E. coli* caused a 10-fold increase in persister formation and an increased tolerance to the antibiotics ampicillin and ofloxacin when compared to the parental strain [12],[50]. Deletion of *glpD* resulted in a decrease of persister formation in the stationary phase, but did not affect tolerance in exponential phase [50]. Deletion of the related gene *glpC* was also found to reduce persistence frequencies compared to the wild type, but this difference was observed only in younger cultures [23].

Finally, genes that regulate heat shock and lipid metabolism have also been implicated in persistence. For example, studies by Vázquez-Laslop et al., demonstrated that the overexpression of heat shock protein DnaJ and lipid A biosynthetic protein PmrC (EptA) from *S. enterica* serovar Typhimurium increased persister formation when cloned into an *E. coli* strain [65]. Conversely, younger cultures of mutant strains lacking *dnaJ* and *surA* (a gene that also encodes a molecular chaperone) had lower persistence frequencies compared to wild type [23]. Therefore, it is clear that, in addition to toxin production and TA modules, bacterial persistence is also linked to the activity of proteins normally dedicated to other functions.

## 6. Future Outlooks

Bacterial persistence is an emerging health threat that causes bacterial infections to be more difficult to treat due to the metabolically inactive state that renders persister cells temporarily tolerant to antibiotic treatment, a condition that contributes to antibiotic resistance. The molecular mechanisms contributing to the persister phenotype are slowly becoming elucidated, as evidenced by the works highlighted in this manuscript. As additional molecular mechanisms contributing to persistence are unraveled, the various molecular pathways that direct persistence can be targeted to prevent persister formation, kill persisters in their dormant state, or even revert persister cells back to their metabolically active, non-persistant state, thus rendering them more vulnerable to antibiotic treatment and preventing recurrent infections. For example, a recent study demonstrated that halogen-containing indoles were capable of eradicating persister formation in biofilms [66]. Another study exposed persister cells to *cis*-decenoic acid, a molecule shown to increase bacterial metabolic activity, which “awakened” the persister cells and led to their loss of tolerance to antibiotics [67],[68]. These and other studies open the door to the potential success of using specific drug combinations (i.e., an “anti-persistence” drug coupled to an effective antibiotic) to treat recalcitrant pathogens known to cause recurrent and persistent infections.

## References

[1] Sengupta S, Chattopadhyay MK, Grossart HP (2013). The multifaceted roles of antibiotics and antibiotic resistance in nature. Front Microbiol.

[2] Centers for Disease Control and Prevention (2013). Antibiotic resistance threats in the United States, 2013.

[3] Abraham EP, Chain E (1940). An enzyme from bacteria able to destroy penicillin. Nature.

[4] Bigger JW (1944). Treatment of staphylococcal infections with penicillin by intermittent sterilisation. Lancet.

[5] Moyed HS, Broderick SH (1986). Molecular cloning and expression of *hipA*, a gene of *Escherichia coli* K-12 that affects frequency of persistence after inhibition of murein synthesis. J Bacteriol.

[6] Centers for Disease Control and Prevention (2016). Newly reported gene, mcr-1, threatens last-resort antibiotics.

[7] Mwangi MM, Wu SW, Zhou Y (2007). Tracking the in vivo evolution of multidrug resistance in *Staphylococcus aureus* by whole-genome sequencing. Proc Natl Acad Sci USA.

[8] Liu B, Pop M (2009). ARDB—Antibiotic Resistance Genes Database. Nucleic Acids Res.

[9] Kussell E, Kishony R, Balaban NQ (2005). Bacterial persistence: a model of survival in changing environments. Genetics.

[10] de Jong IG, Haccou P, Kuipers OP (2011). Bet hedging or not? A guide to proper classification of microbial survival strategies. Bioessays.

[11] Moyed HS, Bertrand KP (1983). *hipA*, a newly recognized gene of *Escherichia coli* K-12 that affects frequency of persistence after inhibition of murein synthesis. J Bacteriol.

[12] Korch SB, Henderson TA, Hill TM (2003). Characterization of the *hipA7* allele of *Escherichia coli* and evidence that high persistence is governed by (p)ppGpp synthesis. Mol Microbiol.

[13] Spoering AL, Lewis K (2001). Biofilms and planktonic cells of *Pseudomonas aeruginosa* have similar resistance to killing by antimicrobials. J Bacteriol.

[14] Keren I, Kaldalu N, Spoering A (2004). Persister cells and tolerance to antimicrobials. FEMS Microbiol Lett.

[15] Mulcahy LR, Burns JL, Lory S (2010). Emergence of *Pseudomonas aeruginosa* strains producing high levels of persister cells in patients with cystic fibrosis. J Bacteriol.

[16] Spoering AL, Vulic M, Lewis K (2006). GlpD and PlsB participate in persister cell formation in *Escherichia coli*. J Bacteriol.

[17] Hofsteenge N, van Nimwegen E, Silander OK (2013). Quantitative analysis of persister fractions suggests different mechanisms of formation among environmental isolates of *E. coli*. BMC Microbiol.

[18] Orman MA, Brynildsen MP (2013). Dormancy is not necessary or sufficient for bacterial persistence. Antimicrob Agents Chemother.

[19] Brouqui P, Bacellar F, Baranton G (2004). Guidelines for the diagnosis of tick-borne bacterial diseases in Europe. Clin Microbiol Infect.

[20] Lewis K (2008). Multidrug tolerance of biofilms and persister cells. Curr Top Microbiol Immunol.

[21] Brooun A, Liu S, Lewis K (2000). A dose-response study of antibiotic resistance in *Pseudomonas aeruginosa* biofilms. Antimicrob Agents Chemother.

[22] Lewis K (2001). Riddle of biofilm resistance. Antimicrob Agents Chemother.

[23] Luidalepp H, Jõers A, Kaldalu N (2011). Age of inoculum strongly influences persister frequency and can mask effects of mutations implicated in altered persistence. J Bacteriol.

[24] Balaban NQ, Merrin J, Chait R (2004). Bacterial persistence as a phenotypic switch. Science.

[25] Jõers A, Kaldalu N, Tenson T (2010). The frequency of persisters in *Escherichia coli* reflects the kinetics of awakening from dormancy. J Bacteriol.

[26] Roostalu J, Jõers A, Luidalepp H (2008). Cell division in *Escherichia coli* cultures monitored at single cell resolution. BMC Microbiol.

[27] Field MF, Lichstein HC (1958). Growth stimulating effect of autoclaved glucose media and its relationship to the CO_2_ requirement of propionibacteria. J Bacteriol.

[28] Rohmer L, Didier H, Miller SI (2011). Are pathogenic bacteria just looking for food? Metabolism and microbial pathogenesis. Trends Microbiol.

[29] Amato S, Brynildsen M (2014). Nutrient transitions are a source of persisters in *Escherichia coli* biofilms. PLoS One.

[30] Kotte O, Volkmer B, Radzikowski JL (2014). Phenotypic bistability in *Escherichia coli*'s central carbon metabolism. Mol Syst Biol.

[31] Radzikowski JL, Vedelaar S, Siegel D (2016). Bacterial persistence in an active σ^S^ stress response to metabolic flux limitation. Mol Syst Biol.

[32] Gentry DR, Hernandez VJ, Nguyen LH (1993). Synthesis of the stationary-phase sigma factor sigma s is positively regulated by ppGpp. J Bacteriol.

[33] Baillie GS, Douglas LJ (1999). *Candida* biofilms and their susceptibility to antifungal agents. Methods Enzymol.

[34] Costerton JW, Stewart PS, Greenberg EP (1999). Bacterial biofilms: a common cause of persistent infections. Science.

[35] Stewart PS (2015). Antimicrobial Tolerance in Biofilms. Microbiol Spectr.

[36] Dalebroux ZD, Swanson MS (2012). ppGpp: magic beyond RNA polymerase. Nat Rev Microbiol.

[37] Maisonneuve E, Castro-Camargo M, Gerdes K (2013). (p)ppGpp controls bacterial persistence by stochastic induction of toxin-antitoxin activity. Cell.

[38] Wood TK, Knabel SJ, Kwan BW (2013). Bacterial persister cell formation and dormancy. Appl Environ Microbiol.

[39] Jayaraman R (2008). Bacterial persistence: some new insights into an old phenomenon. J Biosci.

[40] Black DS, Kelly AJ, Mardis MJ (1991). Structure and organization of *hip*, an operon that affects lethality due to inhibition of peptidoglycan or DNA synthesis. J Bacteriol.

[41] Black DS, Irwin B, Moyed HS (1994). Autoregulation of *hip*, an operon that affects lethality due to inhibition of peptidoglycan or DNA synthesis. J Bacteriol.

[42] Schumacher MA, Piro KM, Xu W (2009). Molecular mechanisms of HipA-mediated multidrug tolerance and its neutralization by HipB. Science.

[43] Hansen S, Vulić M, Min J (2012). Regulation of the *Escherichia coli* HipBA toxin-antitoxin system by proteolysis. PLoS One.

[44] Germain E, Castro-Roa D, Zenkin N (2013). Molecular mechanism of bacterial persistence by HipA. Molecular Cell.

[45] English BP, Hauryliuk V, Sanamrad A (2011). Single-molecule investigation of the stringent response machinery in living bacterial cells. Proc Natl Acad Sci USA.

[46] Keren I, Shah D, Spoering A (2004). Specialized persister cells and the mechanism of multidrug tolerance in *Escherichia coli*. J Bacteriol.

[47] Rotem E, Loinger A, Ronin I (2010). Regulation of phenotypic variability by a threshold-based mechanism underlies bacterial persistence. Proc Natl Acad Sci USA.

[48] Korch SB, Hill TM (2006). Ectopic overexpression of wild-type and mutant *hipA* genes in *Escherichia coli*: effects on macromolecular synthesis and persister formation. J Bacteriol.

[49] Christensen SK, Gerdes K (2003). RelE toxins from *Bacteria* and *Archaea* cleave mRNAs on translating ribosomes, which are rescued by tmRNA. Mol Microbiol.

[50] Spoering AL, Vulic M, Lewis K (2006). GlpD and PlsB participate in persister cell formation in *Escherichia coli*. J Bacteriol.

[51] Pedersen K, Zavialov AV, Pavlov MY (2003). The bacterial toxin RelE displays codon-specific cleavage of mRNAs in the ribosomal A site. Cell.

[52] Zhang Y, Zhang H, Hoeflich KP (2003). MazF cleaves cellular mRNAs specifically at ACA to block protein synthesis in *Escherichia coli*. Mol Cell.

[53] Shah D, Zhang Z, Khodursky AB (2006). Persisters: a distinct physiological state of *E. coli*. BMC Microbiol.

[54] Pandey DP, Gerdes K (2005). Toxin-antitoxin loci are highly abundant in free-living but lost from host-associated prokaryotes. Nucleic Acids Res.

[55] Hu Y, Kwan BW, Osbourne DO (2015). Toxin YafQ increases persister cell formation by reducing indole signaling. Environ Microbiol.

[56] Wang D, Ding X, Rather PN (2001). Indole can act as an extracellular signal in *Escherichia coli*. J Bacteriol.

[57] Aizenman E, Engelberg-Kulka H, Glaser G (1996). An *Escherichia coli* chromosomal “addiction module” regulated by guanosine [corrected] 3′,5′-bispyrophosphate: a model for programmed bacterial cell death. Proc Natl Acad Sci USA.

[58] Reed JL, Vo TD, Schilling CH (2003). An expanded genome-scale model of *Escherichia coli* K-12 (iJR904 GSM/GPR). Genome Biol.

[59] Ren D, Bedzyk LA, Thomas SM (2004). Gene expression in *Escherichia coli* biofilms. Appl Microbiol Biotechnol.

[60] Gonzalez Barrios AF, Zuo R, Hashimoto Y (2006). Autoinducer 2 controls biofilm formation in *Escherichia coli* through a novel motility quorum-sensing regulator (MqsR, B3022). J Bacteriol.

[61] Kasari V, Krug K, Margus T (2010). The *Escherichia coli mqsR* and *ygiI* genes encode a new toxin-antitoxin pair. J Bacteriol.

[62] Li Y, Zhang Y (2007). PhoU is a persistence switch involved in persister formation and tolerance to multiple antibiotics and stresses in *Escherichia coli*. Antimicrob Agents Chemother.

[63] Ma C, SIM S, Shi W (2010). Energy production genes *sucB* and *ubiF* are involved in persister survival and tolerance to multiple antibiotics and stresses in *Escherichia coli*. FEMS Microbiol Lett.

[64] Kwon O, Kotsakis A, Meganathan R (2000). Ubiquinone (coenzyme Q) biosynthesis in *Escherichia coli*: identification of the *ubiF* gene. FEMS Microbiol Lett.

[65] Vázquez-Laslop N, Lee H, Neyfakh AA (2006). Increased persistence in *Escherichia coli* caused by controlled expression of toxins or other unrelated proteins. J Bacteriol.

[66] Lee JH, Kim YG, Gwon G (2016). Halogenated indoles eradicate bacterial persister cells and biofilms. AMB Express.

[67] Mina EG, Marques CNH (2016). Interaction of *Staphylococcus aureus* persister cells with the host when in a persister state and following awakening. Sci Reports.

[68] Marques CNH, Morozov A, Planzos P (2014). The fatty acid signaling molecule *cis*-2-decenoic acid increases metabolic activity and reverts persister cells to an antimicrobial susceptible state. Appl Environ Microbiol.

